# Influence of gender and sexual reproductive state in concentration of CAN f 1 in the fur of dogs (Canis lupus familiaris)

**DOI:** 10.1186/1939-4551-8-S1-A177

**Published:** 2015-04-08

**Authors:** Maicon Paulo, Marconi Rodrigues De Farias, Fábio Nogueira, Michelle Barbosa, L Karla Arruda, Nelson Rosario Filho

**Affiliations:** 1 School of Agricutural Sciences and Veterinary Medicine, Pontifícia Universidade Católica Do Paraná, Brazil; 2 School of Medicine, University of São Paulo, Ribeirão Preto, Brazil; 3 Federal University of Parana, Brazil

## Background

The allergens from cats and dogs have been implicated as extrinsic factors involved in sensitization, precipitation and exacerbation of allergic rhinitis and asthma in susceptible children and adults, at rates ranging from 10 to 25%. The major allergen from the epithelium of dogs is the Can f 1, responsible for the majority of sensitivity reactions to these. The Can f 1 is a lipocalin, which confers adhesive properties, derived from the sebaceous glands and found in fur, scales and saliva of dogs. The aim of this study is to assess the influence of gender and sexual reproductive state on concentrations of Can f 1 in the fur of dogs.

## Methods

Were evaluated 80 healthy household dogs, 40 males (20 whole and 20 neutered) and 40 females (20 whole and 20 neutered), older than one year, regardless of size, weight or breed, free of ectoparasites and sanitized regularly. All samples were separated and sieved resulting in a fine dust used to determine the levels of Can f 1 by enzyme-linked immunosorbent assay (ELISA), using anti can f 1 (Indoor Biothecnologies-Chartottesville USA.) All data were analyzed by ANOVA and Bonferroni method, and the estimated difference between averages for the groups was evaluated by Student's t test, with significance level of 5% (p ≤ 0.05).

## Results

All evaluated dogs had Can f 1 in its fur, and its average concentration was 1.2612±0.67 μg.g^-1^. The concentration of Can f 1 in the fur of dogs was higher in females compared to males (p ≤ 0.05), and there was no difference between neutered and whole (p ≤ 0.05) animals.

## Conclusions

Female dogs have higher amounts of Can f 1 in fur compared to males, and sterilization no influence on their concentrations.

